# Supplementary material for: Custom Gene Panel Analysis Identifies Novel Polymorphisms Associated with Clopidogrel Response in Patients Undergoing Percutaneous Coronary Intervention with Stent
Source: Int J Mol Sci. 2025 Oct 7;26(19):9766. doi: 10.3390/ijms26199766 (PMC12524468; doi:10.3390/ijms26199766)
Supplement: Supplementary file 1 [file ijms-26-09766-s001.zip › ijms-3859360-supplementary.pdf]

Custom Gene Panel Analysis Identifies Novel Polymorphisms Associated with Clopidogrel Response in Patients Undergoing Percutaneous Coronary Intervention with Stent

<u>**Supplementary Figures**</u>

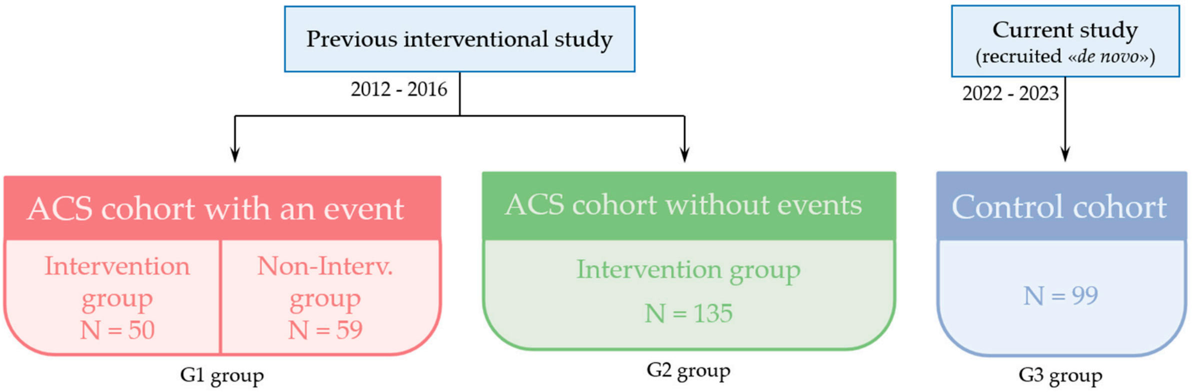

Figure S1. Diagram showing the study groups established in this study to investigate secondary CV effects of antiplatelet therapy.

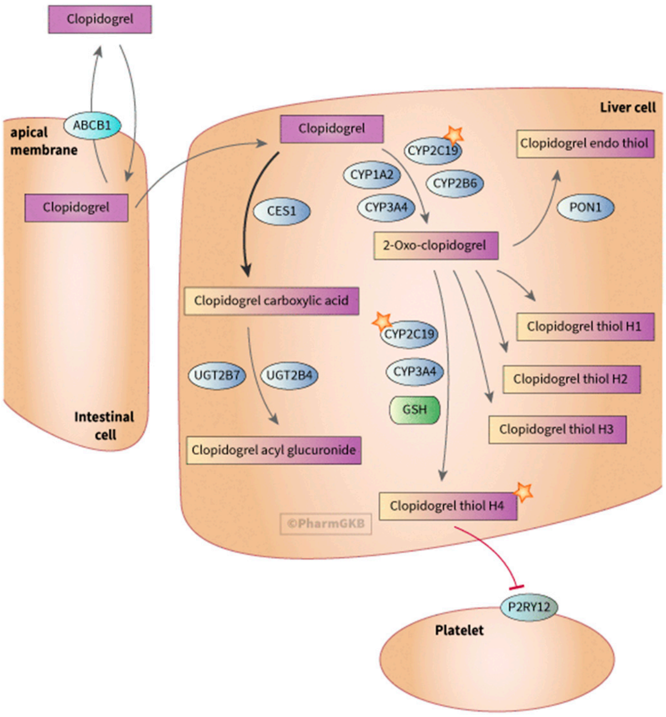

Figure S2. Genes involved in clopidogrel metabolism. Taken from "PharmGKB" website [34].

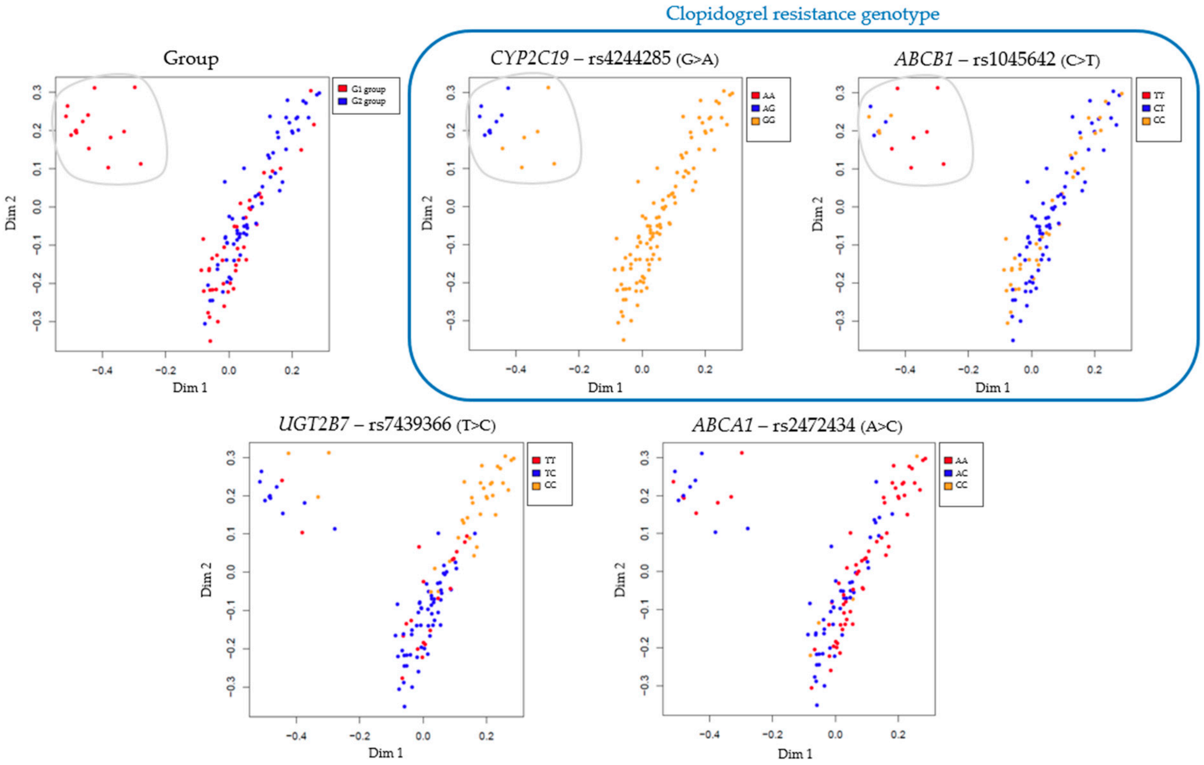

Figure S3. Classical Multidimensional Scaling (MDS) plots in the "event vs non-event" comparison after clopidogrel treatment. The MDS analysis is generated from the "training set" and based on all the variables added in the random forest model. Each plot represents the first two dimensions obtained from the MDS analysis based on the distances between samples collected in the "proximity matrix". The algorithm graphs the samples according to their distance from each other and then applies color according to the variable of interest. The gray circles have been added manually to make the information in the text easier to understand. The X-axis shows a clear separation between a relatively homogeneous cluster of samples belonging to the G1 group (in red) and another, more dispersed cluster containing samples from both G1 and G2 (red and blue). Moreover, it is interesting to note that all clopidogrel-treated individuals with *AG* genotype for *CYP2C19**2 (rs4244285) belong to this G1 subcluster of samples, separated from the rest, as do all samples with *TT* genotype for *ABCB1* c.3435C>T (rs1045642). This clear differentiation suggests that the G1 subgroup corresponds to a very specific set of clopidogrel-resistant individuals, which reinforces the idea that the major source of variability comes from that subgroup of samples, defined by the variable "resistance genotype". In addition to the horizontal segregation, there is a vertical segregation on the Y-axis corresponding to the second major source of variability apparently associated with the rs7439366 (*UGT2B7*) and rs2472434 (*ABCA1*) genotypes. Although these variants did not produce a sharp separation between the G1 and G2 groups, the G2 group appears to be predominantly associated with the *CC* and *AA* genotypes, respectively. The coincidence of these genotypes with the second largest source of variance suggests that combining both markers could improve classification accuracy. Modified from Antúnez-Rodríguez, 2025 [14].

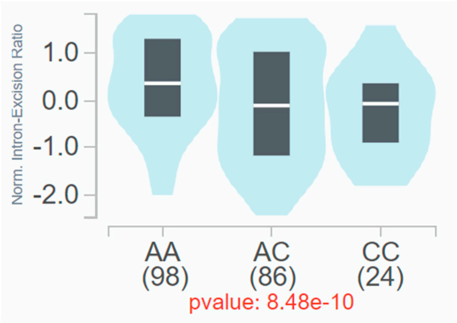

Figure S4. Violin plot showing *ABCA1* expression as a function of rs2472434 genotypes in liver. Taken from Antúnez-Rodríguez, 2025 [14].

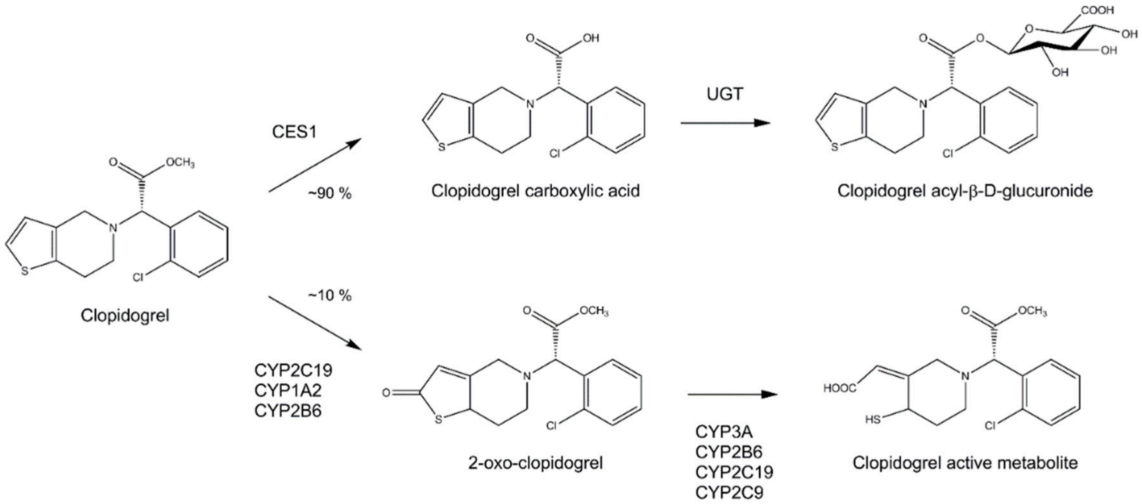

Figure S5. Metabolic pathways of clopidogrel. Clopidogrel is a prodrug that is metabolized by two different pathways. Approximately 10 % of the absorbed dose follows a two-step oxidative metabolism (CYP) pathway to produce the active metabolite cis-5-thiol, while the majority of the dose is metabolized by sequential reactions of hydrolysis to inactive clopidogrel carboxylic acid (CES1) and conjugation to clopidogrel acyl-β-D-glucuronide (UGT). Taken from Kahma *et al*. [26].

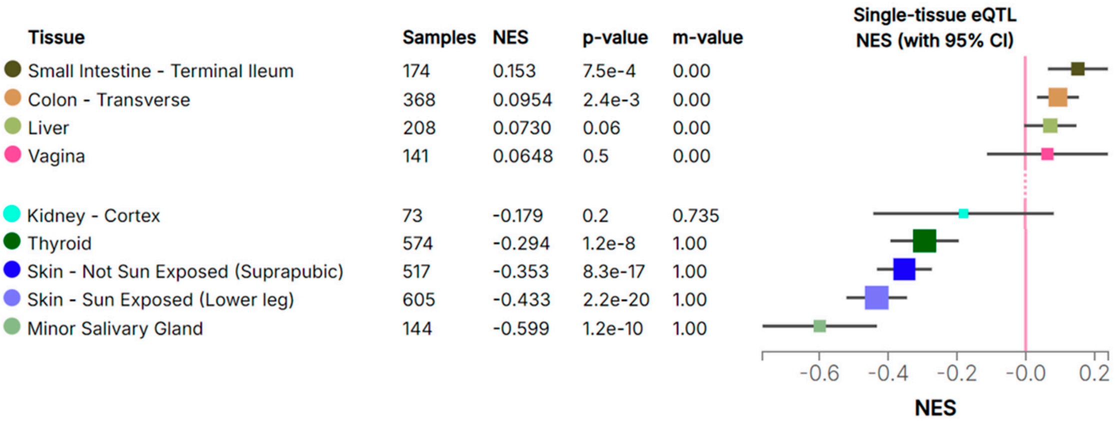

Figure S6. Effect of rs7439366 on *UGT2B7* expression in different tissues. The graph shows the effect size of the rs7439366 variant on *UGT2B7* gene expression in different human tissues. Data obtained from GTEx. Abbreviations: eQTL, expression quantitative trait locus contributing to variation in expression levels; NES, normalized effect size. Taken from Antúnez-Rodríguez, 2025 [14].

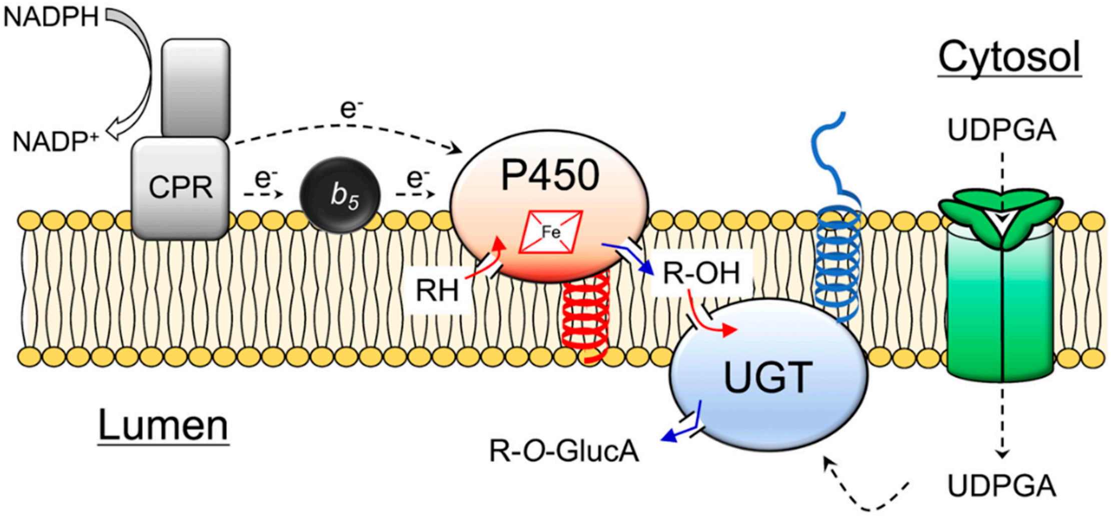

Figure S7. Postulated topology of CYP (P450) and UGT at the endoplasmic reticulum membrane and sequential drug metabolism catalyzed by these enzymes. Taken from Miyauchi *et al*. [27].

Supplementary Introduction

The current study is a continuation of a previous clinical trial conducted by our group from 2012 to 2016 [9, 30], where the target population was patients with ACS undergoing PCI with stenting and indication for antiplatelet therapy (in addition to an indefinite course of acetylsalicylic acid) with a follow-up period of 12 months. Two groups were considered; in the «intervention group», patients carrying *CYP2C19* LoF alleles and/or with *ABCB1 C3435T* homozygous mutant genotype (*TT*) received prasugrel or ticagrelor as antiplatelet therapy, and the remaining patients with normal *CYP2C19* and *ABCB1* gene function received clopidogrel. In the «non-intervention group», patients were treated mainly with clopidogrel. The study concluded that the *CYP2C19/ABCB1* genotype-guided strategy in the choice of antiplatelet therapy was able to reduce MACEs and bleeding rates during the 12 months after PCI compared to a non-guided strategy in ACS-PCI-stent patients. However, the primary endpoint occurred in 32 patients (10.1 %) in the intervention group and in 59 patients (14.1 %) in the non-intervention group (HR 0.63, 95 % CI (0.41–0.97), $p = 0.037$).
From these previous results arose our interest in conducting the current study, we wanted to identify new genetic variants associated with MACEs occurrence during the follow-up period despite PGx-guided antiplatelet therapy.

Supplementary Methods

Random forest analysis

Three models were established including the following "predictor variables" to evaluate their effect on the susceptibility to secondary CV events during the one-year follow-up and to determine their importance as predictors in the classification of patients between the two groups.

- ❖ Clinical variables (for the three models):

Gender, age, BMI.

Principal diagnosis at admission: unstable angina, ST-elevation myocardial infarction or non-ST-elevation myocardial infarction.

Previous CV history: angina, acute myocardial infarction or stroke.

CV risk factors: hypercholesterolemia, hypertension, diabetes, smoking.

Antiplatelet used, β-blockers, statins.

Clopidogrel resistance status, *CYP2C19*2* and *ABCB1* c.3435C>T genotypes.

- ❖ Genetic variables (obtained from association analysis):

«Event vs. non-event» comparison regardless of antiplatelet drug received

rs2472434 (*ABCA1*), rs17618244 (*KLB*), rs114193458 (*LDAH*), rs3827066 (*ZNF335*).

«Event vs. non-event» comparison in patients taking clopidogrel

rs34828128 (*FAM208A*), rs2472434 (*ABCA1*), rs17618244 (*KLB*), rs114193458 (*LDAH*), rs28365062 (*UGT2B7*), rs7439366 (*UGT2B7*), rs11509438 (*GSTO1*), rs10935838 (*P2RY12*), rs1907637 (*P2RY12*), rs28371675 (*CYP2C9*).

«Event vs. non-event» comparison in patients taking prasugrel

rs13047599 (*SON*), rs3732511 (*ARHGEF3*), rs2472434 (*ABCA1*), rs3827066 (*ZNF335*), rs1128503 (*ABCB1*), rs6798347 (*P2RY12*) y rs4891 (*GSTP1*).

Supplemental Results

Two-stage association study

Before proceeding with the association analyses, we would like to clarify one important aspect: although we obtained relatively low *p*-values in the various comparisons performed, none of our results reached the genome-wide significance threshold ($p = 5 \times 10^{-8}$) required to consider these associations truly significant. For this reason, we decided to focus on the variants with the lowest *p*-values, knowing that we could not extrapolate our findings, but could use them to generate hypotheses.

- « Intervention vs. non-intervention » comparison

In this analysis, we compared ACS-PCI-stent patients whose treatment was prescribed based on genetic testing ("intervention group" of the previous study, G1.1 group) with patients with the same condition whose treatment was prescribed based on clinical guidelines without consideration of the genetic profile ("non-intervention group", G1.2 group). In both groups, a MACE and/or hemorrhagic event occurred after antiplatelet therapy, so our objective was to identify whether there was any variant (PGx or CV disease-related) in either group that could have been used to prevent such events.

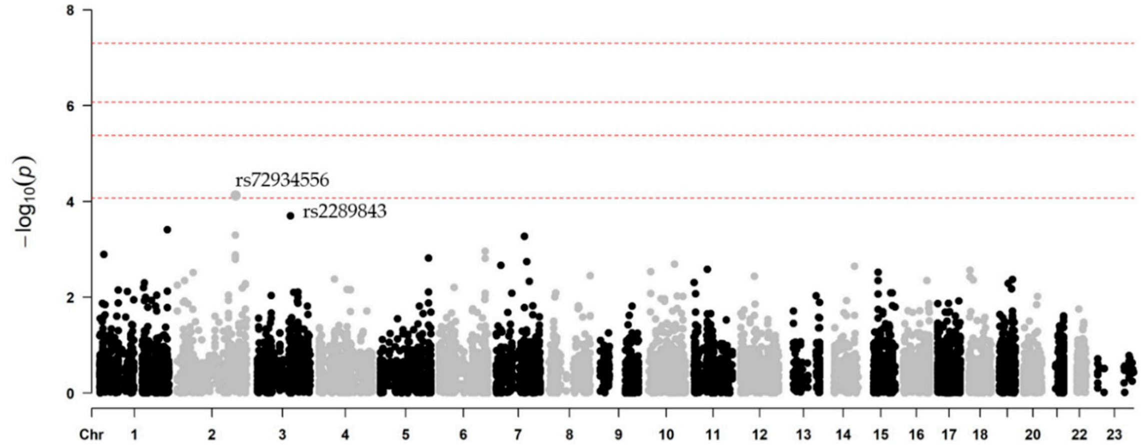

Figure S8. Manhattan plot of association results for the PGx intervention. In this comparison, 109 patients underwent phenotypic association testing. The chromosomal position is on the x-axis and the $-\log_{10}$ of the associated *p*-value is on the y-axis. The four significance levels considered are indicated in dotted lines: genome-wide significance level, $-\log_{10}(5 \times 10^{-8})$; 95 % confidence, $-\log_{10}(0.05/N°rs)$; 90 % confidence, $-\log_{10}(0.1/N°rs)$, and "suggestive", $-\log_{10}(1/N°rs)$. The most significant SNPs in this comparison are labelled. Taken from Antúnez-Rodríguez, 2025 [14].

After association analysis, no genetic variant reached genome-wide significance (5.0×10^{-8}) in the model adjusted for age, gender, and principal components (Figure S8). However, two SNPs were suggestively significant ($p < 1.0 \times 10^{-3}$) (Table S1).

Table S1. Annotation of the top-ranked SNPs identified in the association analysis for the PGx intervention.

SNP	Chr	Position	Ref	Alt	Gene	Func.	MAF	*p*-value	Beta	SD
rs72934556	2	203990789	T	G	*NBEAL1*	synon.	0.12	7.52×10^{-5}	1.5489	0.3912
rs2289843	3	124196094	A	T	*KALRN*	splice reg.*	0.11	2.02×10^{-4}	-1.9878	0.5348
rs2306238	1	237714103	G	A	*RYR2*	intronic	0.21	0.00039	-1.3291	0.3746
rs72932557	2	203846817	A	T	*CARF*	missense	0.13	0.00051	1.3520	0.3890
rs624249	6	160679400	C	A	*SLC22A2*	synon.	0.41	0.00110	0.9859	0.3022
rs6725887	2	203745885	T	C	*WDR12*	intronic	0.12	0.00143	1.1876	0.3724
rs2112703	5	169127097	C	A	*DOCK2*	synon.	0.20	0.00153	-1.0522	0.3321
rs10953541	7	107244545	C	T	*BCAP29*	intronic	0.24	0.00179	1.1782	0.3775
rs11572139	10	96808886	G	A	*CYP2C8*	intronic	0.29	0.00205	-1.1632	0.3773

Genomic position is according to GRCh37/hg19 assembly. MAF was obtained from the Genome Aggregation (gnomAD) - Exomes Database report for Europeans. In this comparison, phenotype-association testing was performed on 109 patients under an additive genetic effect model using the frequentist likelihood score method implemented in the GENESIS v2.30.0 package. Abbreviations: SNP, single nucleotide polymorphism; Chr, chromosome; Ref, reference allele; Alt, alternative allele; Func, functional effect; MAF, minor allele frequency; Beta, beta coefficient corresponding to the effect size measure; SD, standard deviation; synon, synonymous variant. * This variant is annotated as "splice_region_variant&synonymous_variant".
Taken from Antúnez-Rodríguez, 2025 [14].

On chromosome 2, the rs72934556 (c.2871T>G) variant in the *NBEAL1* gene was more common in patients who experienced secondary CV events after conventional antiplatelet therapy ("non-intervention group") compared with those whose treatment was guided by genetic testing (0.27 vs. 0.09; $\beta = 1.55$; $p = 7.52 \times 10^{-5}$). Recent studies suggest that *NBEAL1* plays a protective role against coronary atherosclerosis by regulating cholesterol homeostasis [35]. Therefore, this variant, which is associated with lower gene expression, may increase the risk of coronary artery disease (CAD) in carriers and predispose them to recurrent events. The rs72934556 is also in LD ($r^2 > 0.8$) with two other variants identified in our study: rs72932557 (*CARF* - r^2: 0.84, D': 1.00) and rs6725887 (*WDR12* - r^2: 0.84, D': 1.00), both of which have been associated with atherosclerotic CV disease and genetic risk for recurrent events [36].

Another relevant association was the rs2289843 (c.4098A>T) variant in the *KALRN* gene, which in this case was more common in patients who experienced a secondary CV event after PGx test-guided antiplatelet therapy ("intervention group") compared to the non-intervention group (0.16 vs. 0.03; β = -1.99; $p = 2.02 \times 10^{-4}$). Given the numerous functions of kalirin, including inhibition of inducible nitric oxide synthase activity and action as a guanine nucleotide exchange factor for Rho GTPases, several hypotheses suggest that genetic variation in *KALRN* may contribute to endothelial dysfunction and the progression of atherosclerosis in carriers [37].

- « Event vs. non.event » comparison

In this analysis, we compared ACS-PCI-stent patients who experienced a MACE and/or bleeding after receiving antiplatelet therapy (G1 group) with patients with the same condition who did not experience such events (G2 group). In this case, our objective was to determine whether there was any variant (PGx or CV disease-related) in the G1 group that could be considered to prevent its occurrence.

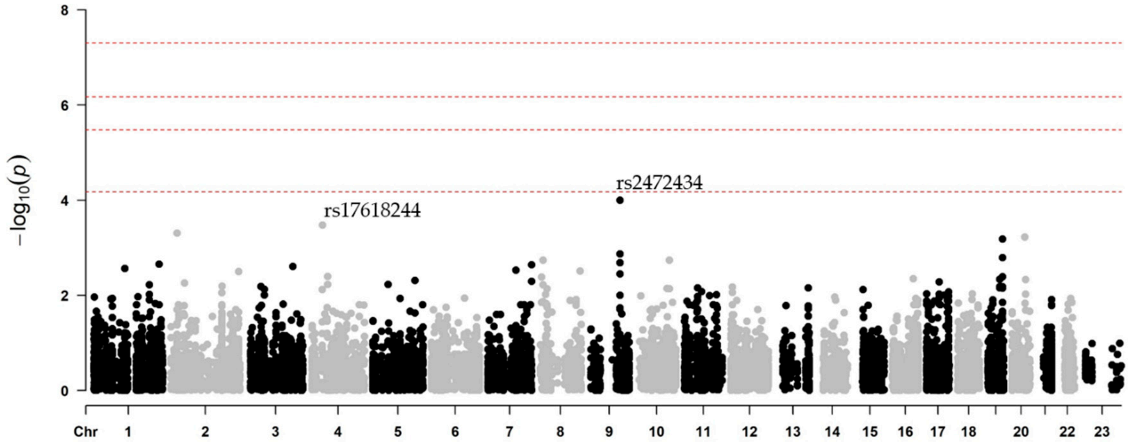

Figure S9. Manhattan plot of association results for development of secondary CV events. In this comparison, 244 subjects underwent phenotypic association testing. The chromosomal position is on the x-axis and the $-\log_{10}$ of the associated *p*-value is on the y-axis. The four significance levels considered are indicated in dotted lines: genome-wide significance level, $-\log_{10}(5 \times 10^{-8})$; 95 % confidence, $-\log_{10}(0.05/N°rs)$; 90 % confidence, $-\log_{10}(0.1/N°rs)$, and "suggestive", $-\log_{10}(1/N°rs)$. The most significant SNPs in this comparison are labeled. Taken from Antúnez-Rodríguez, 2025 [14].

After association analysis, no genetic variant reached genome-wide significance (5.0×10^{-08}) in the model adjusted for age, gender, and principal components (Figure S9). However, three SNPs previously associated with lipid metabolism were found to be suggestively significant (lowest p-value = 1.0×10^{-4}) (Table S2).

The most significant SNP was rs2472434 (c.543+711T>G), an intronic variant in the *ABCA1* gene. Carriers of the alternative *C* allele had a higher incidence of secondary CV events during 1-year follow-up compared with non-carriers (0.35 vs. 0.19; β = -0.92; $p = 1.0 \times 10^{-4}$). In addition, two loci in high LD were identified with this variant, rs2472433 (r^2: 0.97, D': 1.00) and rs2472378 (r^2: 0.87, D': 1.00), which contributed similarly to the increased risk of events ($p = 1.4 \times 10^{-3}$ and $p = 2.0 \times 10^{-3}$, respectively). The *ABCA1* gene plays a key role in regulating cholesterol homeostasis and preventing atherosclerosis. In fact, previous studies have shown that those variants that lead to loss of protein function are associated with reduced cholesterol efflux, promoting intracellular lipid accumulation, which could increase the risk of atherosclerosis and recurrent events in carriers [38, 39].

Table S2. Annotation of the top-ranked SNPs identified in the association analysis for the development of secondary CV events.

SNP	Chr	Position	Ref	Alt	Gene	Func.	MAF	p-value	Beta	SD
rs2472434	9	107623249	A	C	*ABCA1*	intronic	0.28	0.00010	-0.9247	0.2379
rs17618244	4	39448529	G	A	*KLB*	missense	0.19	0.00034	0.8324	0.2321
rs114193458	2	20989039	C	T	*LDAH*	intronic	0.004	0.00049	2.4387	0.6996
rs3827066	20	44586023	C	T	*ZNF335*	intronic	0.17	0.00060	0.8669	0.2526
rs2659122	19	51363026	C	T	*KLK3*	UTR3	0.73	0.00066	0.7246	0.2127
rs2472433	9	107623326	C	T	*ABCA1*	intronic	0.24	0.00136	-0.7751	0.2419
rs2455069	19	51728641	A	G	*CD33*	missense	0.43	0.00162	-0.6172	0.1958
rs35141404	10	112404302	G	A	*RBM20*	synon.	0.19	0.00182	-0.7724	0.2477
rs2409653	8	10677792	T	C	*PINX1*	intronic	0.08	0.00183	1.1410	0.3661
rs2472378	9	107623570	G	T	*ABCA1*	intronic	0.24	0.00206	-0.7452	0.2419

Genomic position is according to GRCh37/hg19 assembly. MAF was obtained from the Genome Aggregation (gnomAD) - Exomes Database report for Europeans. In this comparison, phenotype-association testing was performed on 244 patients under an additive genetic effect model using the frequentist likelihood score method implemented in the GENESIS v2.30.0 package. Abbreviations: SNP, single nucleotide polymorphism; Chr, chromosome; Ref, reference allele; Alt, alternative allele; Func, functional effect; MAF, minor allele frequency; Beta, beta coefficient corresponding to the effect size measure; SD, standard deviation; UTR3, 3′ untranslated region; synon, synonymous variant.
Modified from Antúnez-Rodríguez, 2025 [14].

Another relevant SNP was the missense variant rs17618244 (c.2183G>A, p.Arg728Gln) in the *KLB* gene. In our analysis, carriers of the ancestral *G* allele had a higher incidence of secondary CV events compared with non-carriers ($\beta = 0.83$; $p = 3.4 \times 10^{-4}$). The *KLB* gene encodes the transmembrane β-klotho protein, which is involved in the fibroblast growth factor receptor signaling pathway and contributes to the regulation of bile acid synthesis from cholesterol. Functional studies suggest that the *G* allele of this variant decreases β-klotho stability, which could weaken FGF19-mediated signaling and consequently increase bile acid synthesis in carriers, leading to cardiac dysfunction [40, 41].

Among the major SNPs, we found rs3827066 (c.2442+202G>A), an intronic variant in the *ZNF335* gene, in which carriers of the alternative *T* allele had a lower incidence of secondary CV events compared with non-carriers ($\beta = 0.87$; $p = 6.0 \times 10^{-4}$). This variant is an eQTL (expression quantitative trait loci) associated with increased expression of *PLTP*, a key gene in cholesterol homeostasis and protection against atherosclerosis and potential recurrent events [42, 43].

- « Case vs. control » comparison

In this analysis, we compared ACS-PCI-stent patients (G1&G2 group, which included both event and non-event patients) with a group of controls without structural CV disease (G3 group). In this case, our objective was to determine whether there was any variant in the group of ACS patients that could contribute to the development of CV disease and thus increase the risk of secondary CV events.

After association analysis, no genetic variant reached the genome-wide significance threshold (5.0×10^{-8}) in the model adjusted for age, gender, smoking, and principal components (Figure S10). However, one polymorphism was found to be suggestively significant ($p = 8.7 \times 10^{-5}$) (Table S3).

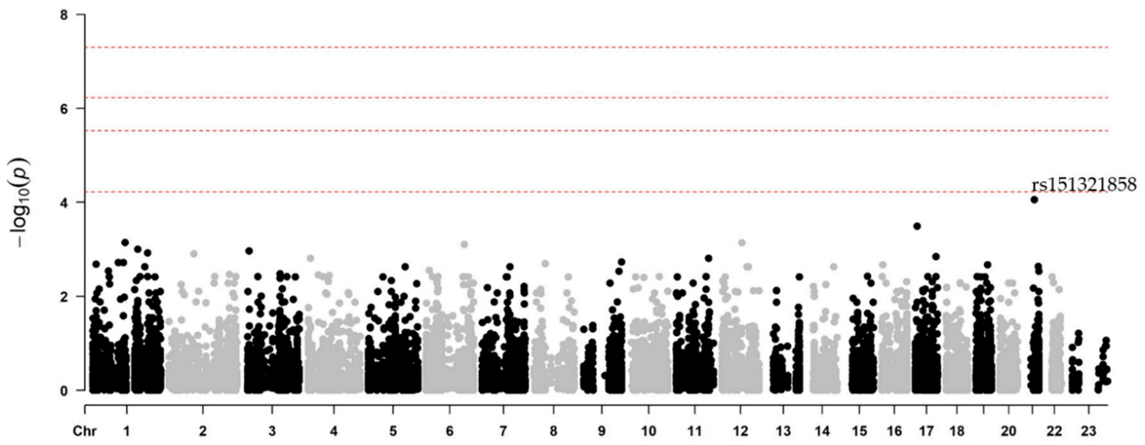

Figure S10. Manhattan plot of association results for the development of CV disease. In this comparison, 343 subjects underwent phenotypic association testing. The chromosomal position is on the x-axis and the $-\log_{10}$ of the associated *p*-value is on the y-axis. The four significance levels considered are indicated in dotted lines: genome-wide significance level, $-\log_{10}(5 \times 10^{-8})$; 95 % confidence, $-\log_{10}(0.05/N°rs)$; 90 % confidence, $-\log_{10}(0.1/N°rs)$, and "suggestive", $-\log_{10}(1/N°rs)$. The most significant SNP in this comparison is labeled. Taken from Antúnez-Rodríguez, 2025 [14].

Table S3. Annotation of the top-ranked SNPs identified in the association analysis for the development of CV disease.

SNP	Chr	Position	Ref	Alt	Gene	Func.	MAF	*p*-value	Beta	SD
rs151321858	21	30699637	A	T	*BACH1*	missense	0.002	8.7×10^{-5}	8.7810	2.2378
rs61804206	1	161658821	A	G	*RPL31P11 ;FCRLA*	intergenic	0.10	0.00099	-1.3171	0.4002
rs11076799	16	4065736	G	A	*ADCY9*	intronic	0.39	0.00213	-0.7167	0.2334
rs5370	6	12296255	G	T	*EDN1*	missense	0.22	0.00282	-0.8039	0.2692
rs79689385	14	64763982	A	G	*ESR2*	intronic	0.04	0.00563	-1.1828	0.4272
rs998488	19	17189468	A	G	*MYO9B*	intronic	0.13	0.00675	-1.1477	0.4236
rs1800566	16	69745145	G	A	*NQO1*	missense	0.19	0.00765	-0.6804	0.2547

Genomic position is according to GRCh37/hg19 assembly. MAF was obtained from the Genome Aggregation (gnomAD) - Exomes Database report for Europeans. In this comparison, phenotype-association testing was performed on 343 subjects under an additive genetic effect model using the frequentist likelihood score method implemented in the GENESIS v2.30.0 package. Abbreviations: SNP, single nucleotide polymorphism; Chr, chromosome; Ref, reference allele; Alt, alternative allele; Func, functional effect; MAF, minor allele frequency; Beta, beta coefficient corresponding to the effect size measure; SD, standard deviation.
Taken from Antúnez-Rodríguez, 2025 [14].

However, upon further analysis of rs151321858 (*BACH1*), it was found that only two individuals in our entire population were heterozygous, and both belonged to the "control group". This finding made it necessary to apply additional prioritization filters. Instead of looking for common variants or variants with a small effect size that could be related to drug metabolism, we focused on identifying CV disease-causing variants that could be responsible for both the development of the disease and the occurrence of adverse events, regardless of the drug administered. For this purpose, "low effect" variants and those with a "score > 0" (more frequent in controls than in cases) were discarded.

Three positions of interest were identified in genes previously associated with CV disease: rs11076799 in *ADCY9* (c.1694-8177C>T), rs5370 in *EDN1* (c.594G>T, p.Lys198Asn) and rs1800566 in *NQO1* (c.559C>T, p.Pro187Ser). As shown in Table S3, ACS patients had a higher frequency of the alternative alleles of these three variants compared to controls without structural CV disease ($\beta < 0$; $p < 0.008$). The *ADCY9* gene encodes an adenylate cyclase involved in cell signaling. Studies in mouse models have shown that *Adcy9* inactivation protects against atherosclerosis by reducing macrophage accumulation in the arterial wall and improving endothelial function [44]. On the other hand, the rs5370 variant in *EDN1*, which encodes a potent vasoconstrictor of the CV system (endothelin-1), has been associated with endothelial dysfunction, inflammation and reduced HDL cholesterol, increasing the risk of CAD in carriers [45, 46]. Finally, regarding the *NQO1* gene, which encodes an antioxidant enzyme (NAD(P)H:quinone oxidoreductase 1), recent studies have associated the missense variant rs1800566 with an increased risk of CAD and atherosclerosis due to reduced or absent enzyme activity [47].

- Secondary CV events after prasugrel treatment

In the study of secondary CV events following prasugrel treatment, we were interested in knowing whether there were genetic variants within the genes described as being involved in prasugrel metabolism that affected the pharmacokinetics and pharmacodynamics of the drug. Therefore, the results of the association analysis were limited to the genes described by PharmGKB (Figure S11).

In this comparison, phenotype-association testing was performed in only 73 patients, so the limited number of prasugrel-treated individuals may have influenced the results and these findings should be interpreted with caution.

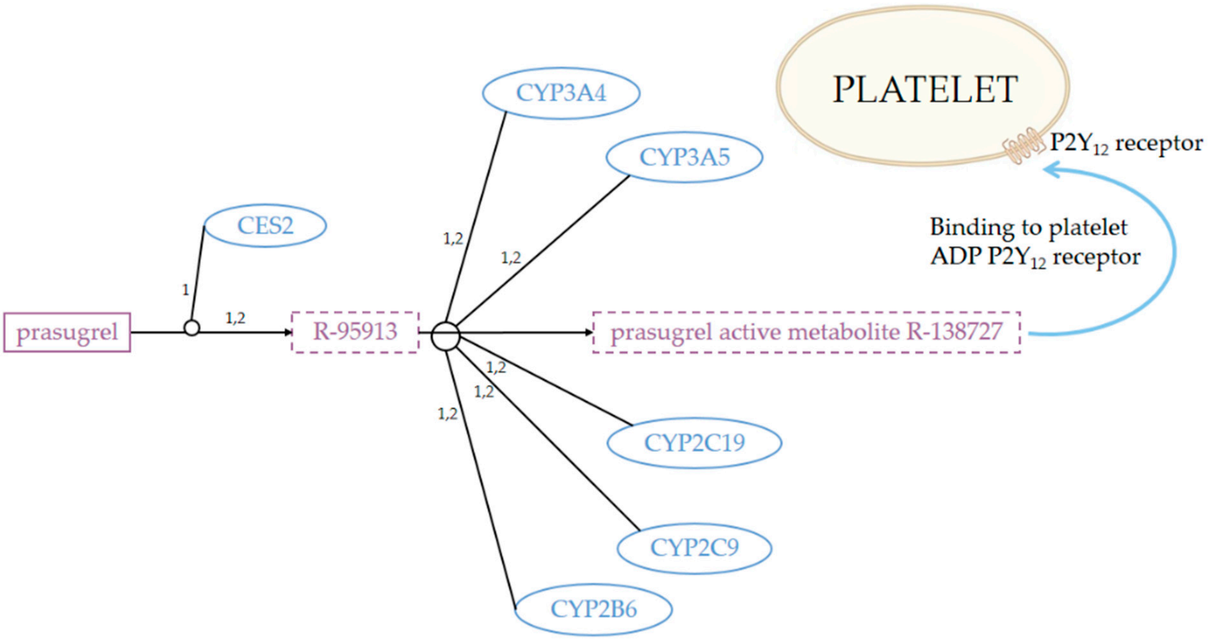

Figure S11. Genes involved in prasugrel metabolism. Taken from "PharmGKB" website [34].

We identified several common variants in the *P2RY12* gene that were suggestively associated with a higher incidence of secondary CV events in prasugrel-treated patients (Table S4). The most significant SNP was rs6798347 (c.-3858C>T), with a higher frequency of the *A* allele in patients with an event compared to those without an event (0.34 vs. 0.12; $\beta = -1.38$; $p = 0.0031$). Interestingly, this variant was not associated with an increased risk of secondary CV events in clopidogrel-treated patients ($p = 0.2296$).

Similarly, the *P2RY12* haplotype consisting of rs6809699, rs2046934 and rs10935838 was associated with an increased risk of secondary CV events in prasugrel-treated patients ($\beta = -1.12$; $p = 0.0248$), whereas the same haplotype was associated with a lower incidence of events in clopidogrel-treated patients ($\beta = 0.66$; $p = 0.0392$). In addition, the rs1907637 variant, which was associated with a lower incidence of events in clopidogrel-treated patients, did not show a significant association in those prescribed prasugrel ($p = 0.08413$).

Table S4. Annotation of the top-ranked SNPs identified in the association analysis for the development of events after prasugrel treatment, limited to genes involved in prasugrel metabolism.

SNP	Chr	Position	Ref	Alt	Gene	Func.	MAF	*p*-value	Beta	SD
rs145425445	19	41518133	G	A	*CYP2B6*	intronic	0.002	0.01682	-3.8537	1.7053
rs6798347	3	151106158	G	A	*P2RY12*	upstream	0.21	0.00306	-1.3835	0.4747
rs6809699	3	151056598	A	C		synon.				
rs2046934	3	151057642	G	A	*P2RY12*	intronic	0.83	0.02485	-1.1209	0.5048
rs10935838	3	151058247	A	G		intronic				
rs4680400	3	151103370	G	A	*P2RY12*	upstream	0.38	0.03189	-0.8758	0.4097
rs7612010	3	151087648	A	G	*P2RY12*	intronic	0.58	0.04839	-0.7429	0.3763
rs1128503	7	87179601	A	G	*ABCB1*	synon.	0.57	0.02383	0.7863	0.3492
rs2235048	7	87138511	G	A	*ABCB1*	intronic	0.47	0.12761	0.5471	0.3591
rs28365062	4	69964271	A	G	*UGT2B7*	synon.	0.14	0.22006	-0.7218	0.5886
rs7439366	4	69964338	T	C	*UGT2B7*	missense	0.48	0.50312	-0.3026	0.4520
rs4244285	10	96541616	G	A	*CYP2C19*	synon.	0.15	0.10404	0.7931	0.4879
rs4986894	10	96522365	T	C		upstream				
rs1907637	3	151104838	A	G	*P2RY12*	upstream	0.87	0.08413	-0.9485	0.5492

Genomic position is according GRCh37/hg19 assembly. MAF was obtained from the Genome Aggregation (gnomAD) - Exomes Database report for Europeans. In this comparison, phenotype-association testing was performed on 73 patients taking prasugrel under an additive genetic effect model using the frequentist likelihood score method implemented in the GENESIS v2.30.0 package. Only genetic variants with significant values ($p < 0.05$) are shown, grouped by gene and ordered by the direction of the pathway shown in Figure S11. From the dotted line, the variants that were significant in the study of secondary CV events after clopidogrel treatment are shown. Abbreviations: SNP, single nucleotide polymorphism; Chr, chromosome; Ref, reference allele; Alt, alternative allele; Func, functional effect; MAF, minor allele frequency; Beta, beta coefficient corresponding to the effect size measure; SD, standard deviation; synon, synonymous variant.
Taken from Antúnez-Rodríguez, 2025 [14].

In addition to genes involved in the prasugrel pathway, we also assessed whether variants previously associated with the development of MACEs and/or hemorrhagic events in clopidogrel-treated patients were replicated in the prasugrel-treated group. None of the variants studied, including rs1128503 in *ABCB1* (in LD with *ABCB1* c.3435C>T; p = 0.1276), rs4244285 in *CYP2C19* (p = 0.1040), and SNPs in *UGT2B7* (rs28365062, p = 0.2201 and rs7439366, p = 0.5031), showed a significant association with the risk of an event in patients receiving prasugrel. However, when the *ABCB1* gene was analyzed, the rs1128503 (c.1446T>C) polymorphism, unlinked to *ABCB1* c.3435C>T, was found to be associated with a lower incidence of secondary CV events in these patients (β = 0.79, p = 0.0238).

Random forest models

- Secondary CV events following prasugrel treatment

Random forest analysis for the "event vs. non-event" comparison in prasugrel-treated patients, as in the overall study regardless of prescribed treatment, revealed that the rs2472434 (*ABCA1*) genotype was the main predictor variable from a ranked list of variables according to their importance in the classification scheme (Figure S12). When the full model was evaluated, the rs2472434 (*ABCA1*) genotype, along with other clinical variables such as principal diagnosis at admission, age, and BMI, retained its relevance in discriminating between patients with and without an event, strengthening its predictive value.

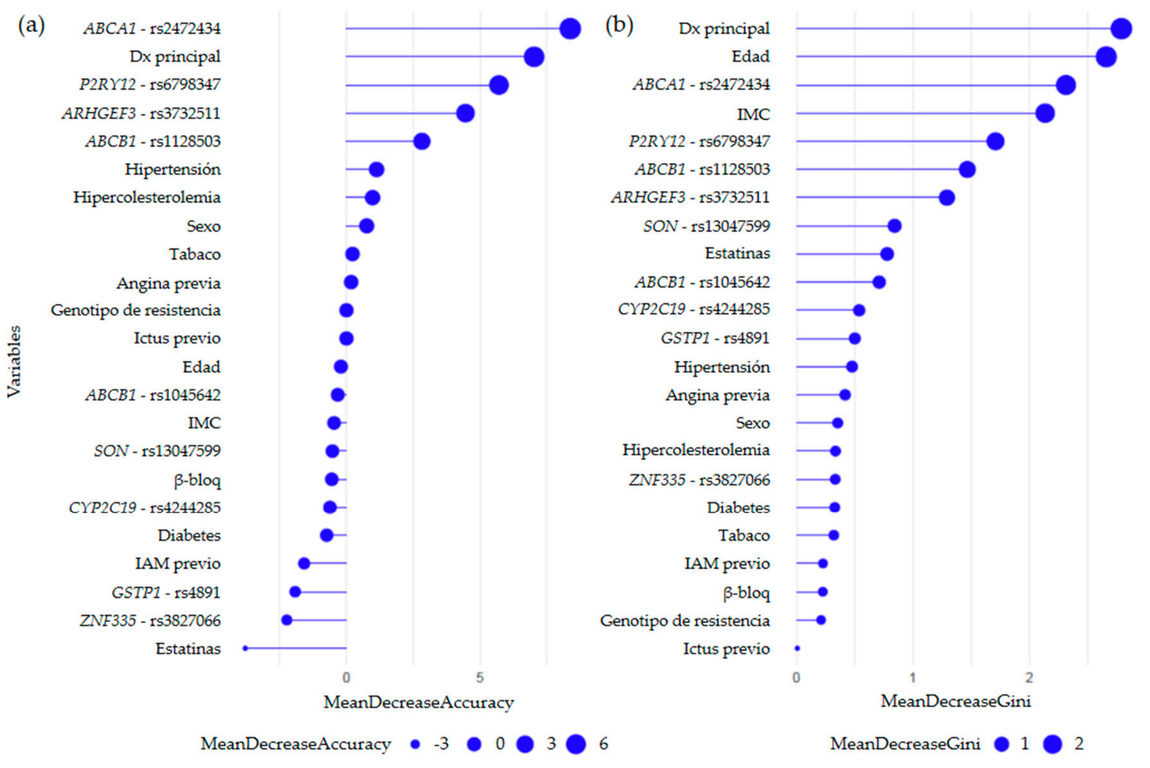

Figure S12. Variable importance plot from the random forest analysis, resulting from a model built around the development of secondary CV events following prasugrel treatment. In this analysis, random forest testing was performed on 73 patients taking prasugrel. Variables are ranked from most to least important in classifying patients with and without events. (**a**) MDA plot, generated from each iteration. (**b**) MDG plot, generated from the entire model rather than each iteration.

The prognostic performance of the variables included in the established model was then evaluated. The ROC curve showed an AUC value of 0.607, indicating moderate discriminatory ability (Figure S13). The model was able to correctly classify 62.5 % of the patients, with a sensitivity of 33.33 % and a specificity of 80 %. Given that the *p*-value obtained was 0.505, the proposed model did not demonstrate good predictive ability to discriminate between prasugrel-treated patients with and without secondary CV events during the follow-up period, which could be attributed to the small sample size of the groups used.

(a)

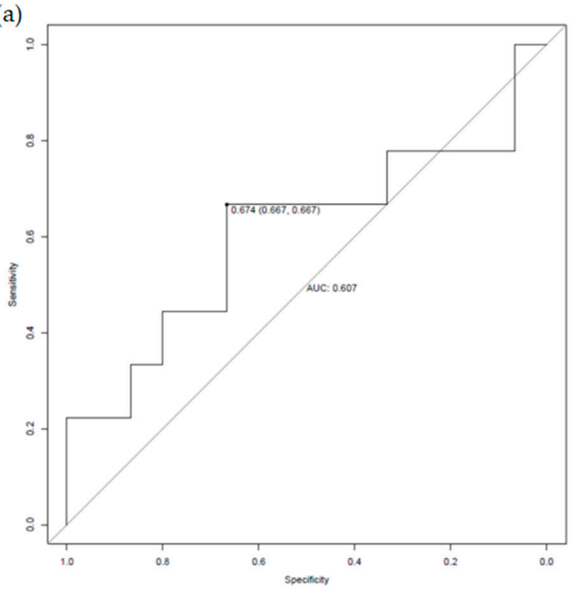

(b)

```
Number of samples used for training the RF model: 49
Number of trees generated: 500. Number of variables used per tree: 5
Number of samples used for testing the RF model: 24
Confusion Matrix and Statistics

          Reference
Prediction case control
   case       3       3
   control    6      12

               Accuracy : 0.625
                 95% CI : (0.4059, 0.812)
    No Information Rate : 0.625
    P-Value [Acc > NIR] : 0.5898

                  Kappa : 0.1429

 Mcnemar's Test P-Value : 0.5050

            Sensitivity : 0.3333
            Specificity : 0.8000
         Pos Pred Value : 0.5000
         Neg Pred Value : 0.6667
             Prevalence : 0.3750
         Detection Rate : 0.1250
   Detection Prevalence : 0.2500
      Balanced Accuracy : 0.5667

       'Positive' Class : case
```

Figure S13. Model performance as established in the "event vs. non-event" comparison after prasugrel treatment. The Random Forest model was trained on 49 samples and tested on 24 samples (total = 73 samples). (**a**) ROC curve with AUC and Youden index statistics, (**b**) parameters used to train the random forest model with the training set and the "confusion matrix" obtained from evaluating the model with the test set. Taken from Antúnez-Rodríguez, 2025 [14].
